# MiR-205 suppressed the malignant behaviors of breast cancer cells by targeting CLDN11 via modulation of the epithelial-to-mesenchymal transition

**DOI:** 10.18632/aging.202988

**Published:** 2021-05-08

**Authors:** Yupeng Shen, Yingchun Xu, Liming Huang, Yongxin Chi, Liwei Meng

**Affiliations:** 1 Medical School of Shaoxing University, Yuecheng, Shaoxing 312000, Zhejiang Province, People’s Republic of China; 2 Department of Breast and Thyroid Surgery, Shaoxing People's Hospital, The First Affiliated Hospital of Shaoxing University, Shaoxing 312000, Zhejiang Province, People’s Republic of China

**Keywords:** breast cancer, miR-205, CLDN11, proliferation

## Abstract

Some Aberrant expression of miRNAs plays an important role in the occurrence and distant metastasis of breast cancer. This study aimed to identify crucial miRNA signatures for breast cancer using microarray data from the Gene Expression Omnibus database, including ductal carcinoma *in situ* and invasive duct carcinoma. In this study, we founded that miR-205 was significantly down-regulated in breast cancer, and the low expression of miR-205 was significantly associated with the TNM stage of breast cancer. *In vitro,* functional studies revealed that over-expression of miR-205 inhibited the proliferation and promoted apoptosis of breast cancer cells MDA-MB-231. Mechanistically, claudin 11 (CLDN11) was found to be the direct target of miR-205; the function of miR-205 could be exerted via downregulation of the target gene CLDN11 in breast cancer cells. Furthermore, the over-expression of miR-205 promoted the expression of the epithelial marker E-cadherin but reduced the mesenchymal markers in breast cancer cells. These results collectively indicated the tumor-suppressive role of miR-205 in breast cancer by targeting CLDN11; implying miR-205 may serve as a novel therapeutic target for breast cancer.

## INTRODUCTION

Globally, breast cancer represents the most commonly diagnosed malignancy affecting women. In 2020, an estimated 2.3 million new cases indicated that one in every 8 cancers diagnosed was breast cancer worldwide. It is also the leading cause of cancer-associated mortality, with an estimated 684,996 deaths from breast cancer in 2020 [1–5]. At present, there are many treatments for breast cancer, including surgery, chemotherapy, radiotherapy, endocrine therapy and molecular targeted therapy, but the mortality of breast cancer is still very high. Technological advances in breast cancer have significantly improved the understanding of breast cancer biology [6–9]; however, the precise molecular mechanism driving breast cancer remains elusive [10–12]. Therefore, investigating the molecular mechanism related to breast cancer is crucial for enhanced understanding of the pathogenesis for the early detection and precise treatment of breast cancer.

MicroRNA (miRNA) is an endogenous non coding single stranded RNA with a length of 18-22 NT. 50% of the known miRNAs are located in tumor related gene regions/fragile sites, suggesting that miRNAs can act as carcinogenic or tumor suppressor genes to promote or inhibit cancer. Studies have shown that miRNAs regulate post transcriptional gene expression mainly through the degradation or translation inhibition of target gene mRNAs, and the imbalance and/or mutation of miRNAs can cause tumorigenesis [13–19]. Many miRNAs can play a dual role in tumor both as tumor suppressors and oncogenes by targeting corresponding tumor suppressor genes or oncogenes [20]. Moreover, dysregulation of miRNA expression has been associated with cancer onset, progression, and metastasis. In cancer, tumor suppressor miRNAs act as antagonists based on their capability to repress oncogenes, resulting in the occurrence and development of various malignant tumors such as breast cancer. Furthermore, dysregulation of miRNA is predominantly involved in the proliferation and metastasis of triple-negative breast cancer through epithelial-mesenchymal transformation (EMT), which may be useful in the diagnosis, therapy assessment, and prognosis of breast cancer. Therefore, investigating the target regulatory function of miRNA can provide a new insight into the clinical treatment of breast cancer [21, 22].

Accumulating evidence has indicated the roles of tumor-derived miRNAs in breast cancer. In this context, Youness et al. revealed that in TNBC, knockout of sONE, a newly discovered lncRNA, resulted in a significant decrease in the expression of tumor suppressor *TP53* and an increase in the expression of oncogenic transcription factor c-myc. Moreover, sONE affected the progression of TNBC by altering the expression levels of a panel of its downstream tumor suppressor miRNAs, including miR-34a, miR-15, miR-16, and let-7a [23–26]. Mohammadi-Yeganeh et al. demonstrated that miR-381 targets primary genes of the Wnt signaling pathway and inhibit the development of TNBC; besides, overexpression of miR-381 can reduce the migration and invasion potential of MDA-MB-231 cells; at the same time, overexpression of miR-381 can inhibit the capability of lung and liver metastasis and prolong the survival in murine TNBC model [27, 28].

An increasing number of studies have confirmed that miR-205 is associated with the occurrence of a variety of tumors, and its expression status is different in different tumor types, such as low expression has been found in prostate cancer; conversely, the significantly high expression has been detected in endometrial cancer, non-small cell lung cancer, head and neck squamous cell carcinoma, suggesting that miR-205 may function as a tumor suppressor or oncogenic miRNA in specific tissues [29–31]. However, the precise molecular mechanism and function of miR-205 in breast cancer remains obscure. In this study, we investigated the potential regulatory role of miR-205 in breast cancer occurrence and development. Using gain- and loss-of-function assays, we further confirmed the downstream target genes regulated by miR-205 both *in vivo* and *in vitro*.

## RESULTS

### Differential expression miRNA profile in human breast cancer

**Figure 1:**
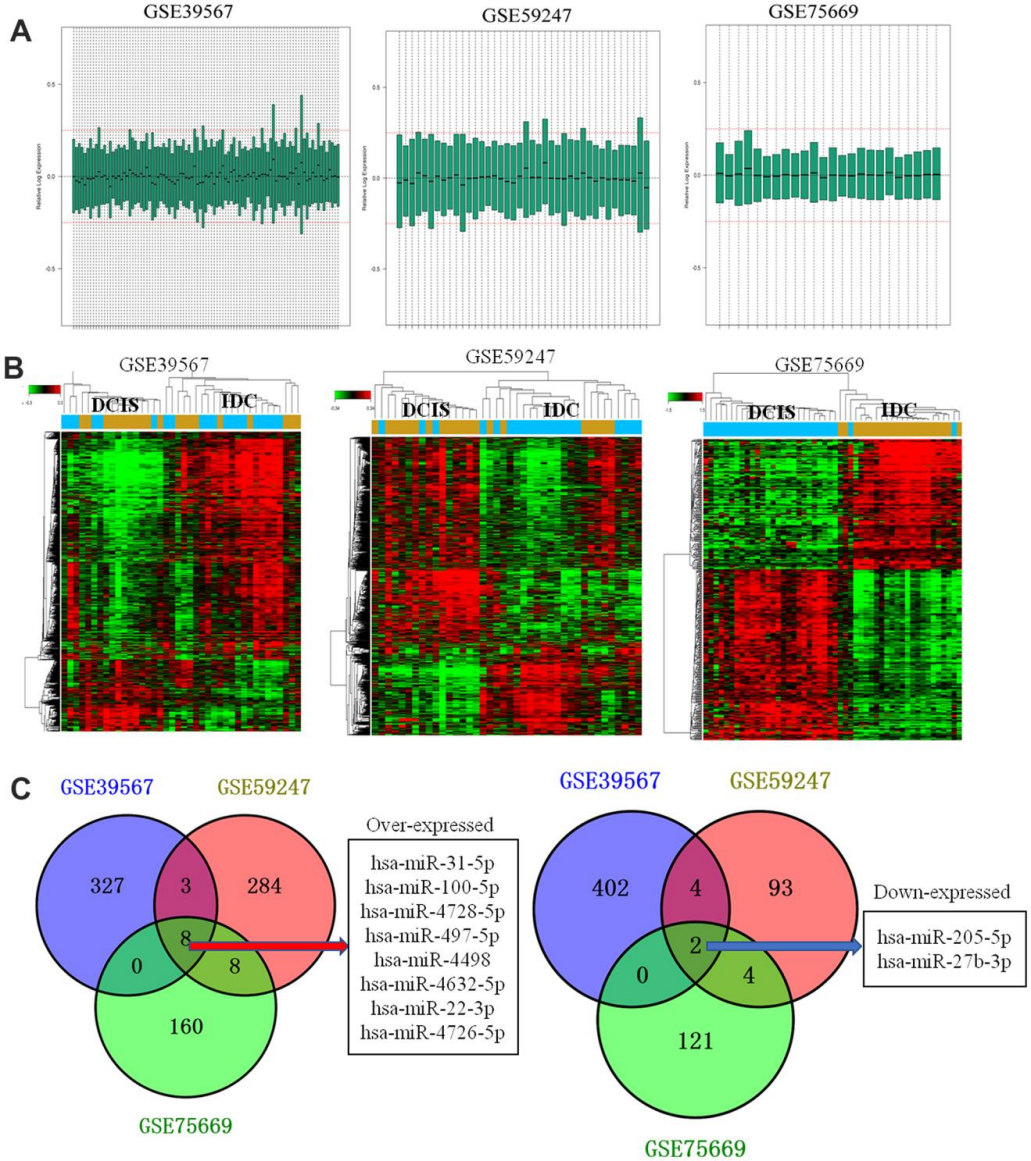
**Data processing and identification of differentially expressed miRNAs.** (**A**) Data processing results after normalization of GSE39567, GSE59247, and GSE75669; (**B**) Heat map showing differentially expressed miRNAs in ductal carcinoma *in situ* (DCIS) and invasive ductal carcinoma (IDC) samples of GSE39567, GSE59247, and GSE75669; Red and green indicated the high and lower expression, respectively. (**C**) Venn diagram. The overlapped differentially expressed miRNAs between different datasets were analyzed.

**Figure 2:**
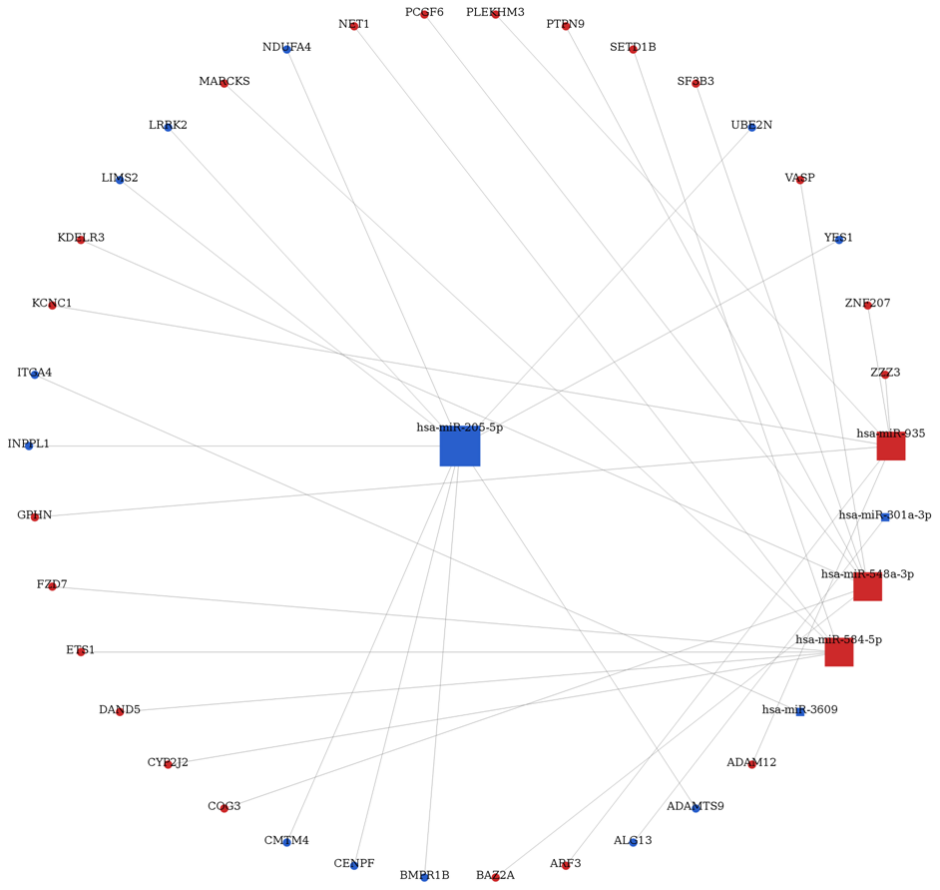
Differently expressed miRNAs-mRNA interaction network in breast cancer.

After normalization (Figure 1A), a total of 311,245 and 224 DEMs were identified in GSE39567, GSE59247, and GSE75669 (Figure 1B) datasets, respectively. In comparison, 8 (Figure 1A) and 2 (Figure 1B) DEMs overlapped among the three analyzed datasets.

### DEMs-mRNAs interaction network

Using the miRwalk 2.0 database, as presented in Figure 1C, which 4234 interactions were predicted for the DEMs based on their target genes and downstream molecular pathways. Notably, 56 target genes for 11 DEMs were extracted to construct the regulatory network of differentially expressed miRNA and target genes (Figure 2). In this regulatory network, hsa-miR-205 was specifically and consistently expressed in two datasets and interacts with CLDN11 (claudin 11). Thus, miR-205 was predicted to interact with CLDN11.

### Function enrichment for target genes of DEMs

**Figure 3:**
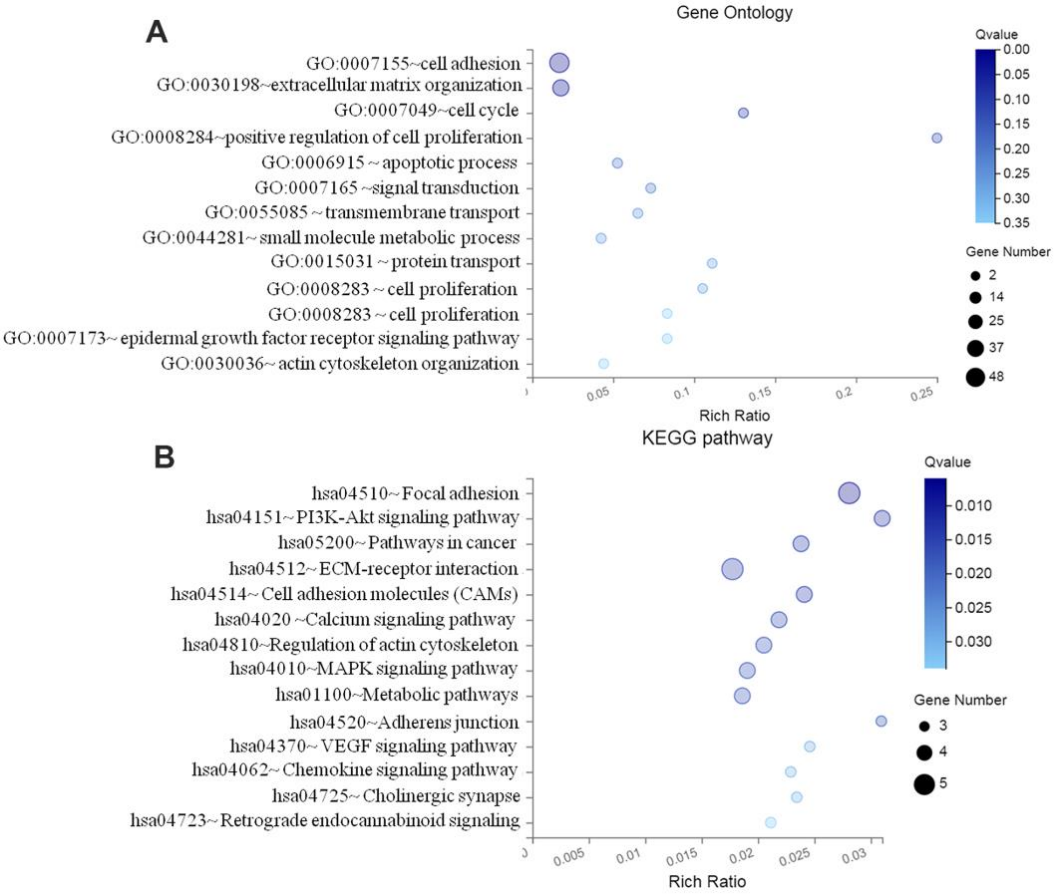
**Functional enrichment analyses of differentially expressed miRNA target genes.** (**A**) GO enrichment analysis; (**B**) KEGG pathway enrichment analysis GO, Gene ontology; KEGG, Kyoto Encyclopedia of Genes and Genomes; FDR, false discovery rate.

Through DAVID online tool, GO terms and KEGG analyses of the identified target genes were performed, as presented in Figure 3, to predict their potential functions. The result indicated that the top 13 BP (biology process) for all the target genes of DEMs were mostly involved in cell adhesion (GO:0007155;CLDN11, E-cadherin), extracellular matrix organization (GO:0030198;CLDN11, MMP2), GO:0007049- cell cycle (CDK2, CDK4), and positive regulation of cell proliferation (GO:0008284;TCM2, CDKN1B) (Figure 3A). 11 signaling pathways were further enriched, including hsa04510-Focal adhesion (CLDN11, SMAD4), hsa04512-ECM-receptor interaction (MMP9, ECM1), and hsa05200-pathways in cancer (CLDN11, MTMR14) (Figure 3B).

### Validation of crucial miRNA using additional datasets

**Figure 4:**
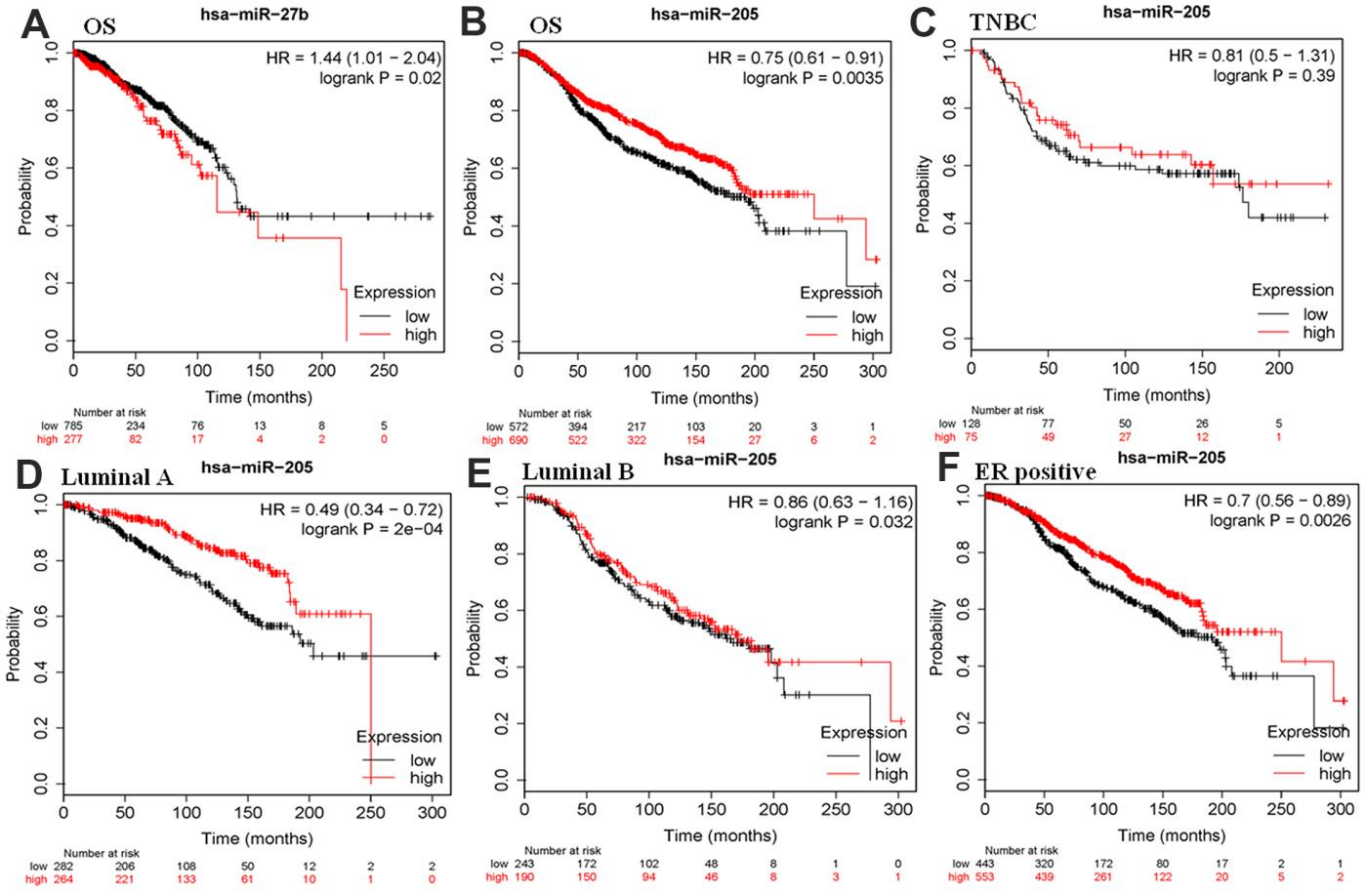
**Kaplan–Meier plotter analysis was used to assess the correlation of miR-27b and miR-205 with survival for patients with breast cancer in the METABRIC database.** HR, hazard ratio; (**A**, **B**) OS, overall survival; (**C**) TNBC, triple-negative breast cancer; (**D**) luminal A, (ER+HER2-KI67-); (**E**) luminal A; (**F**) ER, estrogen receptor.

Previously, our bioinformatics analysis suggested that the low expression of miR-27b and miR-205 may involve in breast cancer development. Single factor regression analysis was performed to investigate whether they were associated with survival using the METABRIC data [32, 33]. Kaplan-Meier plotter database was used to assess their associations with the prognosis of breast cancer. The results suggested that downregulation of has-miR-27b was identified as a risk factor for poor prognosis, which was contrary to our expected results (Figure 4A). Moreover, miR-205 served as a protective factor for a favorable prognosis (Figure 4B). We further investigated the association of miR-205 with various subtypes of breast cancer. However, there was no correlation between the expression of miR-205 and the survival and prognosis in three negative breast cancer patients (Figure 4C). Furthermore, we found that over-expression of miR-455-5p independently predicted the poor prognosis in luminal A (ER+HER2-KI67-), luminal B, and ER-positive subtypes (Figure 4D–4F).

### Low expression of miR-205 in breast cancer tissues and cells

**Figure 5:**
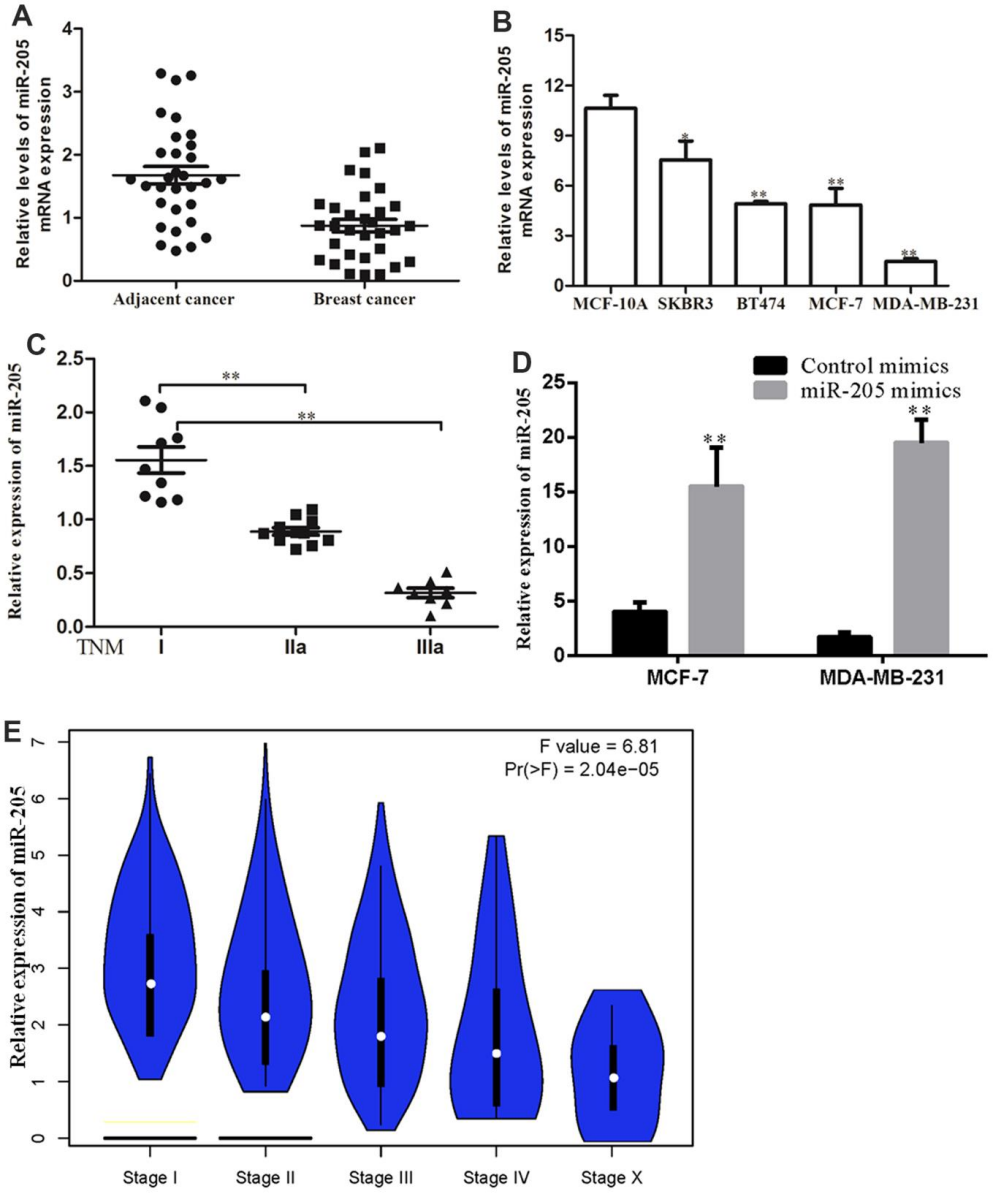
**miR-205 expression was downregulated in breast cancer.** (**A**) Expression levels of miR-205 in breast cancer tissues and adjacent normal breast tissues as analyzed by the qRT-PCR assay. (**B**) The expression of miR-205 in breast cancer cell lines and normal cell MCF10A. (**C**) The expression of miR-205 in breast cancer patients with TNM stages was significantly reduced. (**D**) Both MCF7 and MDA-MB-231 cells were transfected with control miRNA or miR-205 mimics, and the expression of miR-205 was confirmed by qRT-PCR. (**E**) The association between expression of miR-205 and clinical characteristics of patients with breast cancer. LinkedOmics database with TCGA data was used for this analysis. All experiments were carried out in triplicate. Data were expressed as mean ± standard deviation (SD).

The result of qRT-PCR revealed that the expression of miR-205 was significantly downregulated in breast cancer tissues as compared with the normal tissues (Figure 5A). Consistently, the expression level of miR-205 in MCF-10A was higher than that in breast cancer cell (MCF-7, SK-BR3, T47D and MDA-MB-231). Among the four breast cancer cell lines, SK-BR3 had the highest expression and MDA-MB-231 had the lowest expression (Figure 5B). As indicated in Figure 5C, the low expression of miR-205 is closely related to the malignant degree of breast cancer. In order to further study the effect of miR-205 on the biological function of breast cancer cells, we chose to overexpress miR-205 in MDA-MB-231 cells and silence miR-205 in MCF-7 cells (Figure 5D). Furthermore, we also analyzed the expression of miR-205 in breast cancer patients based on the TCGA database. Compared with normal breast tissue, the expression of miR-205 was significantly lower in breast cancer tissue, and was similar to that in normal breast tissue (Figure 5E).

### Over-expression of miR-205 inhibited the proliferation and sphere formation of breast cancer cells

**Figure 6:**
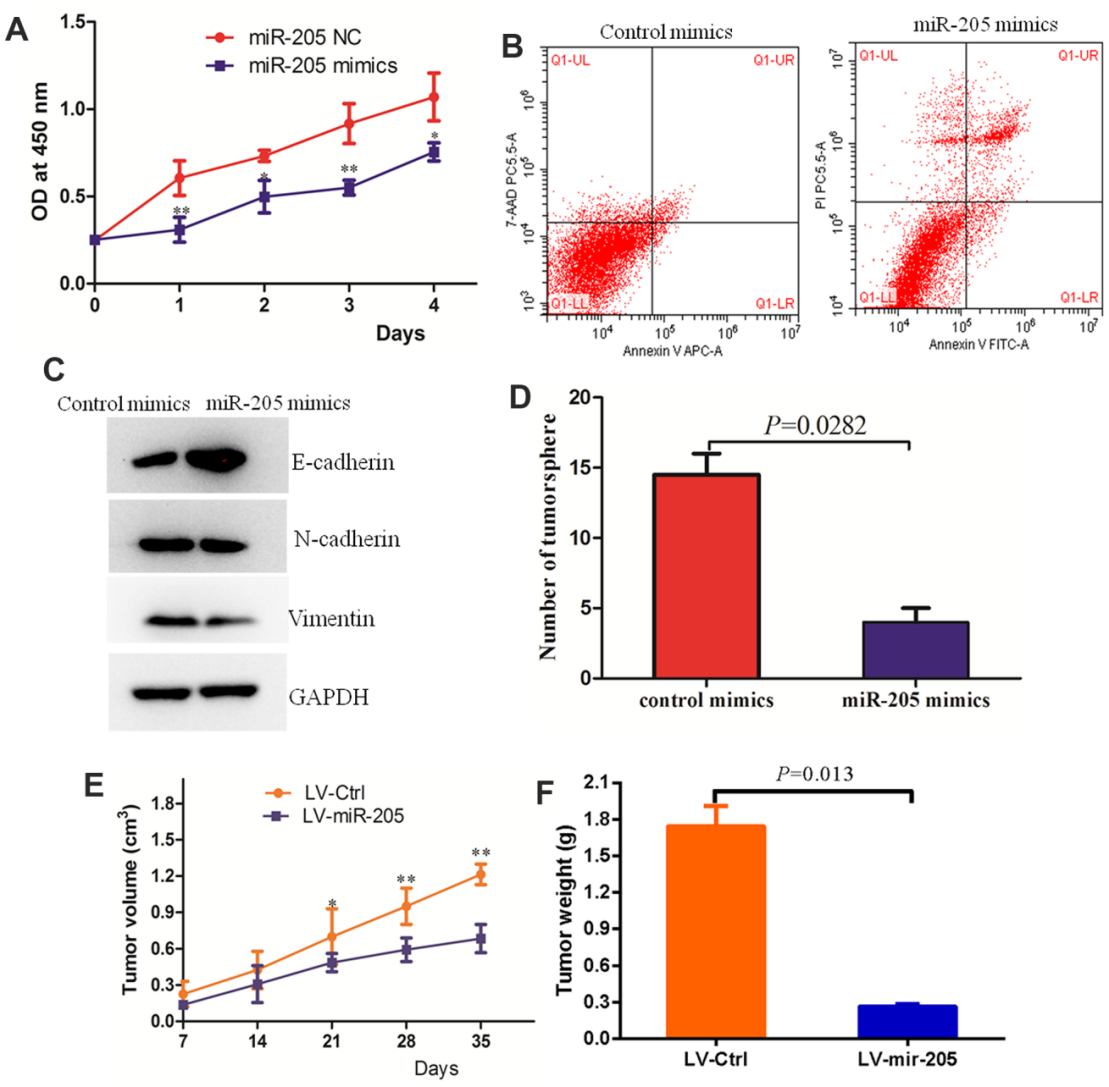
**Over-expression of miR-205 inhibited the malignant behaviors of breast cancer cells.** (**A**) The proliferation of MCF7 and MDA-MB-231 cells with the transfection of control miRNA or miR-205 mimic as determined by the CCK-8 assay. (**B**) Over-expression of miR-205 promoted the apoptosis of MDA-MB-231 cells. (**C**) Over-expression of miR-205 regulated the expression of biomarkers of EMT in MDA-MB-231 cells. (**D**) Over-expression of miR-205 significantly inhibited the colony formation of breast cancer cells. (**E**) The tumor volume of the over-expressed miR-205 group was lower than the control group. (**F**) The tumor weight of the over-expressed miR-205 group was lower than the control group.*P<0.05, **P<0.01.

In order to determine whether miR-205 can affect the proliferation of MDA-MB-231 cells, we overexpressed mir-205 in MDA-MB-231 cells and detected the cell growth by CCK8 assay. Compared with the negative control group, the proliferation of MDA-MB-231 cells in miR-205group was significantly inhibited (Figure 6A). Flow cytometry data indicated that over-expression of miR-205 promoted cell apoptosis compared with the control cells (Figure 6B). Epithelial mesenchymal transition (EMT) not only plays a key role in the development process, but also participates in tissue healing, organ fibrosis and carcinogenesis. We found that over-expression of miR-205 caused an increase in the level of E-cadherin (epithelial marker) in MDA-MB-231 cells, but a decrease in N-cadherin (mesenchymal marker) in MDA-MB-231 by immunoblotting and immunofluorescence assays (Figure 6C and Supplementary Figure 2). Moreover, over-expression of miR-205 inhibited the EMT of breast cancer cells. The results of tumor microsphere formation indicated that over-expression of miR-205 significantly inhibited the colony formation of MDA-MB-231 compared with the control group (Figure 6D). To further validate the effect of miR-205 over-expression on breast cancer *in vivo*, tumor xenograft models were established using 4-week-old BALB/c female nude mice inoculated with stable miR-205 (LV-miR-205) overexpressing cells and control cells (LV-Ctrl) in the bilateral armpit. The qRT-PCR analysis confirmed that the miR-205 expression levels were significantly over-expressed in MDA-MB-231 cell lines (Supplementary Figure 1). Compared to the control group, the tumors formed by LV-miR-205 breast cancer cells developed relatively slowly, with significantly smaller size and lighter weight (Figure 6E, 6F). In conclusion, these results suggest that miR-205 plays a role as a tumor-suppressor in the progression of breast cancer.

### CLDN11 was a target of miR-205 in breast cancer cells

**Figure 7:**
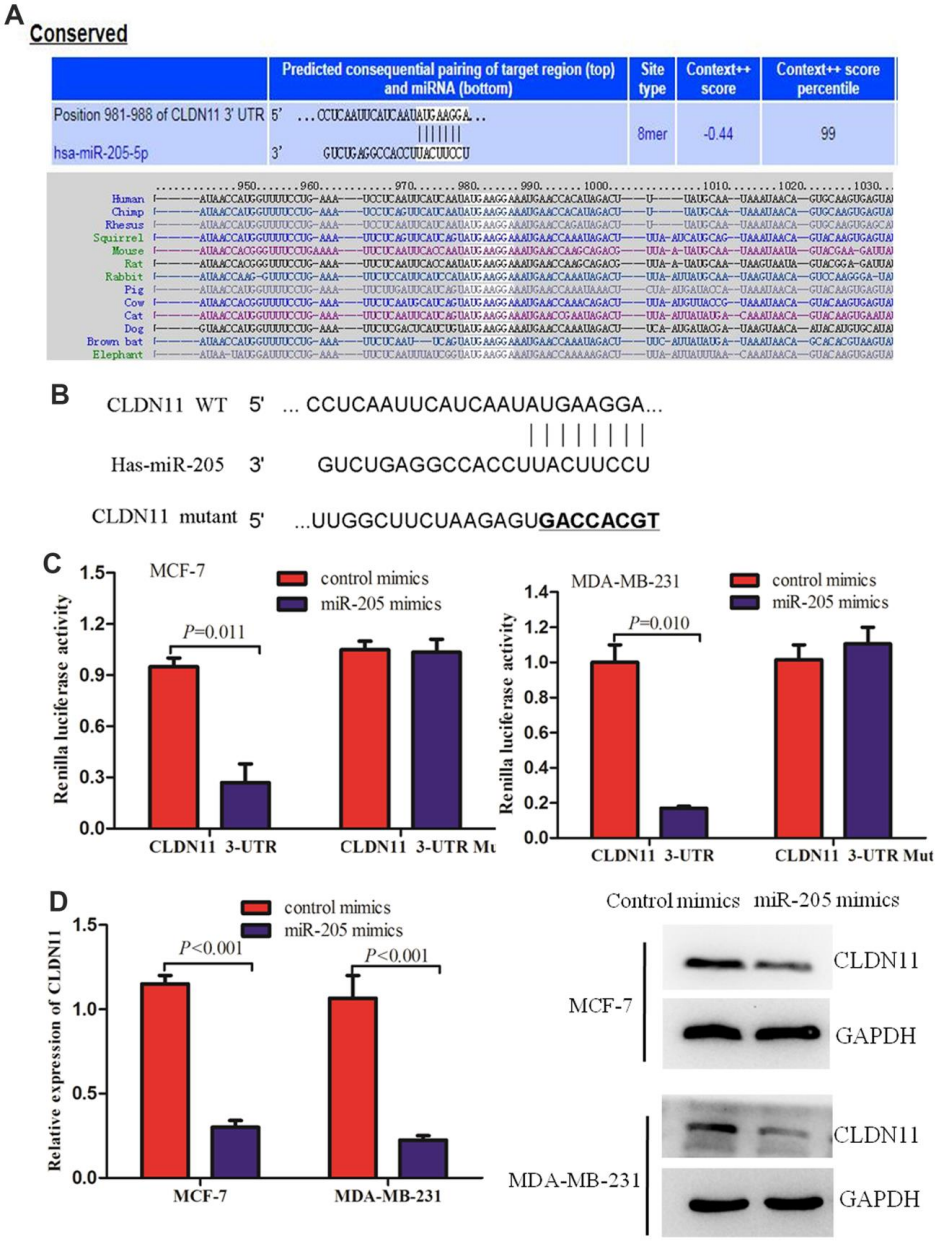
**CLDN11 was a direct target of miR-205.** (**A**, **B**) The predicted binding sites of miR-205 at the 3’-UTR of CLDN11 using bioinformatics (**B**, **C**) Overexpression of miR-205 reduced the luciferase activity of wild-type 3’-UTR of CLDN11 in MCF-7 and MDA-MB-231 cells. (**D**) Over-expression of miR-205 suppressed both the mRNA and protein levels of CLDN11 in both MCF7 and MDA-MB-231 cells.

In order to explore the molecular biological mechanism of miR-205 downstream, miRDB online and Targetscan were used to predict the target genes regulated by miR-205. The data showed miR-205 has a high score in the scoring system, suggesting that its expression may be regulated by miR-205 (Figure 7A). Furthermore, fluorescence *in situ* hybridization (FISH) was performed to detect miR-205 distribution and subcellular fractionation, and identified miR-205 was predominantly localized in the cytoplasm in MDA-MB-231 cells (Supplementary Figure 2). To further confirm the regulatory ability of miR-205 on CLDN11, we co transfected MDA-MB-231 cells with the constructed fluorescent reporter plasmids PmirGLO-miR-205-CLDN11 WT and PmirGLO-miR-205-CLDN11 MUT, miR-205 mimics and mimics control respectively, and detected the activity of dual fluorescent reporter genes 48 hours later. The results showed that in MDA-MB-231 cells, miR-205 significantly inhibited the activity of wild-type CLDN11 3 '- UTR (*P*< 0.05), but did not inhibit the activity of mutant CLDN11 3'–UTR (Figure 7B, 7C). Together, these results indicated that CLDN11 could be the putative target of miR-205-5p. Our previous work demonstrated that CLDN11 may function as a prognostic biomarker and could serve as an important new therapeutic target for human breast cancer.

To further investigate whether the binding of miR-205 with the 3’-UTR of CLDN11 affected the mRNA stability of CLDN11, the mRNA level of CLDN11 was detected by RT-qPCR of cells transfected with miR-205. The results indicated that overexpression of miR-205 significantly reduced the mRNA level of CLDN11 in breast cancer cells (Figure 7D). Consistent with this result, Western blot analysis also revealed that over-expression of miR-205 suppressed the protein level of CLDN11 in both MCF7 and MDA-MB-231 cells (Figure 7D). Collectively, these findings demonstrated that CLDN11 was a direct target of miR-205 in breast cancer cells.

## DISCUSSION

In China, the incidence rate of breast cancer is increasing year by year. More than 30 women are diagnosed with breast cancer every year. The incidence rate of breast cancer is especially high in the city of eastern coastal areas and economically developed cities. From the age of onset, the incidence rate of breast cancer has gradually increased since the age of 20, and reached a high value at 45~50 years old. With the popularity of new treatment strategies and methods, the global mortality of breast cancer is gradually decreasing. However, in China, especially in the vast rural areas, the declining trend of breast cancer mortality is not significant [34, 35]. miRNAs represent a class of small, 18- to 28-nucleotide-long, noncoding regulatory RNAs that function by binding to the 3’-UTR of target mRNAs, leading to degradation or translation inhibition of target mRNAs [19, 36]. Several deregulated miRNAs have been involved in a variety of cellular processes, including cell proliferation, differentiation, and apoptosis [20, 37, 38]. Numerous studies indicated that miRNAs could play a dual role as tumor suppressors or oncogenes in tumorigenicity, depending on tissue type and specific targets [39]. These pieces of evidence suggested that miRNAs may serve as preferred therapeutic targets for cancer therapy.

In this study, we provided evidence that miR-205 was down-regulated in breast cancer. The expression level and regulation of miRNA in breast cancer were markedly different. The expression of miR-205 was significantly downregulated in comprehensive miRNA expression profiling. Consistently, Wu et al. used miRNA primer design and probe technology to study miRNA expression profile in breast cancer and found that the expression of miR-205 in breast cancer tissue was 80% lower than that in normal breast tissue. Furthermore, the present study also confirmed that the low expression of miR-205 was inversely associated with the TNM stage of breast cancer. Besides, decreased expression of miR-205 was associated with the tumor progression in breast cancer patients. However, over-expression of miR-205 inhibited cell growth via targeting CLDN11.

In summary, this study identified that miR-205 was significantly downregulated in breast cancer, and the low expression of miR-205 was inversely associated with the TNM stage of breast cancer. However, over-expression of miR-205 inhibited the proliferation and promoted apoptosis of breast cancer cells MDA-MB-231. Mechanistically, claudin 11 (CLDN11) was found to be the direct target of miR-205; the function of miR-205 could be exerted via down regulation of the target gene CLDN11 in breast cancer cells. Taken together, these findings indicated the tumor-suppressive role of miR-205 in breast cancer by targeting CLDN11; implying miR-205 may serve as a novel therapeutic target for breast cancer.

## MATERIALS AND METHODS

### Identification of target genes of DEMs DEMs-mRNAs interaction network

The limma package of R language was used to screen the differential miRNAs from the miRNA expression profile data. MiRNAs with log2fc absolute value greater than 1 and P < 0.05 were selected as miRNAs with significant differences for subsequent analysis. Mirtabase database (http://mirtarbase.cuhk.edu.cn/) mainly collects miRNA target genes verified by experiments, and the latest update is in June 2019. Using cluster profiler package in R, the predicted target genes were analyzed for KEGG and GO enrichment.

### RNA isolation and quantitative real-time polymerase chain reaction (qRT-PCR)

Total mRNA was extracted from tissues and cells using Eastep® Super Total RNA Extraction Kit (Promega, LS1040, MA, USA). According to the manufacturer's protocol, miRNAs were extracted from the cells using a miRcute miRNA isolation kit (FP401, TianGEN, Beijing, China). In brief, the cells were lysed with lysis buffer. Then, chloroform was added and shaken for 1 min. After resting the cells for 5 min at room temperature, the cell was centrifuged for 20 min (13400 g), and miRNA solution was collected, washed with 75%ethanol (E801077, Macklin) and centrifuged at 13400g for 15 min. The miRNA pellet was resuspended in RNase-free water. Then, PrimeScript™RT reagent Kit with gDNA Eraser (Takara, Dalian, China) was used to reverse-transcribe RNAs into cDNAs according to the manufacturer's instructions. Quantitative real-time PCR (qRT-PCR) was used to quantify the expression of miR-205, using Power SYBR Green PCR Master cDNA obtained on a 7500 real-time RT-PCR system. Sequences of sense and antisense primers used to detect CLDN11 were as follows: CLDN11 forward, 5'-CGGTGTGGCTAAGTACAGGC-3'; CLDN11 reverse, 5'-CGCAGTGTAGTAGAAACGGTTTT-3'; GAPDH and U6 RNA were used as the endogenous control. The relative gene expression levels were determined with the 2−^ΔΔCT^ method.

### Cell counting kit-8 (CCK-8) assays

Cell proliferation was assessed using the Cell Counting Kit (CCK-8) assay (Bosterbio, China). After the transfection of miR-205 mimics or control, 1.0×10^4^ cells were seeded into 96-well plates containing cells 100 μl complete medium [40]. After incubation with 10 μ l CCK for 30 minutes at 37° C, the absorbance was measured at 450 nm using a microplate reader [41]. All experiments were independently performed in triplicate.

### Statistical analysis

All data were expressed as mean ± standard deviation (SD). Comparison between two groups was performed with the Student's *t* test. One-way analysis of variance (ANOVA) followed by Dennett's test was used for multiple groups. Statistical analyses were performed with SPSS 17.0 software version (SPSS, Inc., Chicago, IL, USA). *P*<0.05 was considered to be statistically significant.

## Supplementary Material

Supplementary Figures

## References

[1] Siegel RL, Miller KD, Jemal A. Cancer statistics, 2020. CA Cancer J Clin. 2020; 70:7–30. 10.3322/caac.21590 31912902

[2] DeSantis CE, Miller KD, Dale W, Mohile SG, Cohen HJ, Leach CR, Goding Sauer A, Jemal A, Siegel RL. Cancer statistics for adults aged 85 years and older, 2019. CA Cancer J Clin. 2019; 69:452–67. 10.3322/caac.21577 31390062 PMC12103238

[3] Fillon M. Perioperative management may lead to less pain after breast cancer surgery. CA Cancer J Clin. 2019; 69:5–6. 10.3322/caac.21465 30475405

[4] DeSantis CE, Ma J, Gaudet MM, Newman LA, Miller KD, Goding Sauer A, Jemal A, Siegel RL. Breast cancer statistics, 2019. CA Cancer J Clin. 2019; 69:438–51. 10.3322/caac.21583 31577379

[5] Barton MK. Symptoms, cancer-related distress, and overall distress may contribute to racial disparities in the outcomes of patients with early-stage breast cancer. CA Cancer J Clin. 2017; 67:257–58. 10.3322/caac.21371 28548681

[6] Giuliano AE, Connolly JL, Edge SB, Mittendorf EA, Rugo HS, Solin LJ, Weaver DL, Winchester DJ, Hortobagyi GN. Breast Cancer-Major changes in the American Joint Committee on Cancer eighth edition cancer staging manual. CA Cancer J Clin. 2017; 67:290–303. 10.3322/caac.21393 28294295

[7] Curigliano G, Loibl S. CDK4/6 inhibitors in breast cancer: one more step towards reduced mortality. Lancet Oncol. 2020; 21:191–92. 10.1016/S1470-2045(19)30808-3 31859247

[8] Novel ADC Solidifies Role in Breast Cancer. Cancer Discov. 2020; 10:167. 10.1158/2159-8290.CD-NB2019-139 31843763

[9] Schmid P, Abraham J, Chan S, Wheatley D, Brunt AM, Nemsadze G, Baird RD, Park YH, Hall PS, Perren T, Stein RC, Mangel L, Ferrero JM, et al. Capivasertib Plus Paclitaxel Versus Placebo Plus Paclitaxel As First-Line Therapy for Metastatic Triple-Negative Breast Cancer: The PAKT Trial. J Clin Oncol. 2020; 38:423–33. 10.1200/JCO.19.00368 31841354

[10] Allison KH, Hammond ME, Dowsett M, McKernin SE, Carey LA, Fitzgibbons PL, Hayes DF, Lakhani SR, Chavez-MacGregor M, Perlmutter J, Perou CM, Regan MM, Rimm DL, et al. Estrogen and Progesterone Receptor Testing in Breast Cancer: ASCO/CAP Guideline Update. J Clin Oncol. 2020; 38:1346–66. 10.1200/JCO.19.02309 31928404

[11] Pisano ED. AI shows promise for breast cancer screening. Nature. 2020; 577:35–36. 10.1038/d41586-019-03822-8 31894156

[12] Cristofanilli M. Time for a shift in molecular down staging in luminal breast cancer. Lancet Oncol. 2020; 21:2–3. 10.1016/S1470-2045(19)30806-X 31838014

[13] Mori MA, Ludwig RG, Garcia-Martin R, Brandão BB, Kahn CR. Extracellular miRNAs: From Biomarkers to Mediators of Physiology and Disease. Cell Metab. 2019; 30:656–73. 10.1016/j.cmet.2019.07.011 31447320 PMC6774861

[14] Clancy JW, Zhang Y, Sheehan C, D’Souza-Schorey C. An ARF6-Exportin-5 axis delivers pre-miRNA cargo to tumour microvesicles. Nat Cell Biol. 2019; 21:856–66. 10.1038/s41556-019-0345-y 31235936 PMC6697424

[15] Liu Z, Wang J, Cheng H, Ke X, Sun L, Zhang QC, Wang HW. Cryo-EM Structure of Human Dicer and Its Complexes with a Pre-miRNA Substrate. Cell. 2018; 173:1191–1203.e12. 10.1016/j.cell.2018.03.080 29706542

[16] Soengas MS, Hernando E. TYRP1 mRNA goes fishing for miRNAs in melanoma. Nat Cell Biol. 2017; 19:1311–12. 10.1038/ncb3637 29087386

[17] Kanchan RK, Siddiqui JA, Mahapatra S, Batra SK, Nasser MW. microRNAs Orchestrate Pathophysiology of Breast Cancer Brain Metastasis: Advances in Therapy. Mol Cancer. 2020; 19:29. 10.1186/s12943-020-1140-x 32059676 PMC7023699

[18] Wu HJ, Hao M, Yeo SK, Guan JL. FAK signaling in cancer-associated fibroblasts promotes breast cancer cell migration and metastasis by exosomal miRNAs-mediated intercellular communication. Oncogene. 2020; 39:2539–49. 10.1038/s41388-020-1162-2 31988451 PMC7310603

[19] Zhang J, Liu B, He J, Ma L, Li J. Inferring functional miRNA-mRNA regulatory modules in epithelial-mesenchymal transition with a probabilistic topic model. Comput Biol Med. 2012; 42:428–37. 10.1016/j.compbiomed.2011.12.011 22245099

[20] Wu H, Zhu S, Mo YY. Suppression of cell growth and invasion by miR-205 in breast cancer. Cell Res. 2009; 19:439–48. 10.1038/cr.2009.18 19238171 PMC2664859

[21] Persson H, Søkilde R, Häkkinen J, Pirona AC, Vallon-Christersson J, Kvist A, Mertens F, Borg Å, Mitelman F, Höglund M, Rovira C. Frequent miRNA-convergent fusion gene events in breast cancer. Nat Commun. 2017; 8:788. 10.1038/s41467-017-01176-1 28983113 PMC5629207

[22] Pichiorri F, Palmieri D, De Luca L, Consiglio J, You J, Rocci A, Talabere T, Piovan C, Lagana A, Cascione L, Guan J, Gasparini P, Balatti V, et al. Correction: *In vivo* NCL targeting affects breast cancer aggressiveness through miRNA regulation. J Exp Med. 2017; 214:1557. 10.1084/jem.2012095001172017c 28104811 PMC5413319

[23] Ahmed Youness R, Amr Assal R, Mohamed Ezzat S, Zakaria Gad M, Abdel Motaal A. A methoxylated quercetin glycoside harnesses HCC tumor progression in a TP53/miR-15/miR-16 dependent manner. Nat Prod Res. 2020; 34:1475–80. 10.1080/14786419.2018.1509326 30526087

[24] ElKhouly AM, Youness RA, Gad MZ. MicroRNA-486-5p and microRNA-486-3p: Multifaceted pleiotropic mediators in oncological and non-oncological conditions. Noncoding RNA Res. 2020; 5:11–21. 10.1016/j.ncrna.2020.01.001 31993547 PMC6971376

[25] Shaalan YM, Handoussa H, Youness RA, Assal RA, El-Khatib AH, Linscheid MW, El Tayebi HM, Abdelaziz AI. Destabilizing the interplay between miR-1275 and IGF2BPs by Tamarix articulata and quercetin in hepatocellular carcinoma. Nat Prod Res. 2018; 32:2217–20. 10.1080/14786419.2017.1366478 28817968

[26] Youness RA, Hafez HM, Khallaf E, Assal RA, Abdel Motaal A, Gad MZ. The long noncoding RNA sONE represses triple-negative breast cancer aggressiveness through inducing the expression of miR-34a, miR-15a, miR-16, and let-7a. J Cell Physiol. 2019; 234:20286–97. 10.1002/jcp.28629 30968427

[27] Menbari MN, Rahimi K, Ahmadi A, Mohammadi-Yeganeh S, Elyasi A, Darvishi N, Hosseini V, Abdi M. miR-483-3p suppresses the proliferation and progression of human triple negative breast cancer cells by targeting the HDAC8>oncogene. J Cell Physiol. 2020; 235:2631–42. 10.1002/jcp.29167 31508813

[28] Menbari MN, Rahimi K, Ahmadi A, Elyasi A, Darvishi N, Hosseini V, Mohammadi-Yeganeh S, Abdi M. MiR-216b-5p inhibits cell proliferation in human breast cancer by down-regulating HDAC8 expression. Life Sci. 2019; 237:116945. 10.1016/j.lfs.2019.116945 31605710

[29] Zhong G, Xiong X. miR-205 promotes proliferation and invasion of laryngeal squamous cell carcinoma by suppressing CDK2AP1 expression. Biol Res. 2015; 48:60. 10.1186/s40659-015-0052-5 26515287 PMC4625464

[30] Zhu M, Wang X, Gu Y, Wang F, Li L, Qiu X. MEG3 overexpression inhibits the tumorigenesis of breast cancer by downregulating miR-21 through the PI3K/Akt pathway. Arch Biochem Biophys. 2019; 661:22–30. 10.1016/j.abb.2018.10.021 30389444

[31] Su N, Qiu H, Chen Y, Yang T, Yan Q, Wan X. miR-205 promotes tumor proliferation and invasion through targeting ESRRG in endometrial carcinoma. Oncol Rep. 2013; 29:2297–302. 10.3892/or.2013.2400 23589079

[32] Milioli HH, Vimieiro R, Riveros C, Tishchenko I, Berretta R, Moscato P. The Discovery of Novel Biomarkers Improves Breast Cancer Intrinsic Subtype Prediction and Reconciles the Labels in the METABRIC Data Set. PLoS One. 2015; 10:e0129711. 10.1371/journal.pone.0129711 26132585 PMC4488510

[33] Curtis C, Shah SP, Chin SF, Turashvili G, Rueda OM, Dunning MJ, Speed D, Lynch AG, Samarajiwa S, Yuan Y, Gräf S, Ha G, Haffari G, et al, and METABRIC Group. The genomic and transcriptomic architecture of 2,000 breast tumours reveals novel subgroups. Nature. 2012; 486:346–52. 10.1038/nature10983 22522925 PMC3440846

[34] Picon-Ruiz M, Morata-Tarifa C, Valle-Goffin JJ, Friedman ER, Slingerland JM. Obesity and adverse breast cancer risk and outcome: Mechanistic insights and strategies for intervention. CA Cancer J Clin. 2017; 67:378–97. 10.3322/caac.21405 28763097 PMC5591063

[35] Greenlee H, DuPont-Reyes MJ, Balneaves LG, Carlson LE, Cohen MR, Deng G, Johnson JA, Mumber M, Seely D, Zick SM, Boyce LM, Tripathy D. Clinical practice guidelines on the evidence-based use of integrative therapies during and after breast cancer treatment. CA Cancer J Clin. 2017; 67:194–232. 10.3322/caac.21397 28436999 PMC5892208

[36] Krell J, Stebbing J, Frampton AE, Carissimi C, Harding V, De Giorgio A, Fulci V, Macino G, Colombo T, Castellano L. The role of TP53 in miRNA loading onto AGO2 and in remodelling the miRNA-mRNA interaction network. Lancet. 2015 (Suppl 1); 385:S15. 10.1016/S0140-6736(15)60330-0 26312837

[37] de Rie D, Abugessaisa I, Alam T, Arner E, Arner P, Ashoor H, Åström G, Babina M, Bertin N, Burroughs AM, Carlisle AJ, Daub CO, Detmar M, et al, and FANTOM Consortium. An integrated expression atlas of miRNAs and their promoters in human and mouse. Nat Biotechnol. 2017; 35:872–78. 10.1038/nbt.3947 28829439 PMC5767576

[38] Ng EK, Wong CL, Ma ES, Kwong A. MicroRNAs as New Players for Diagnosis, Prognosis, and Therapeutic Targets in Breast Cancer. J Oncol. 2009; 2009:305420. 10.1155/2009/305420 19644558 PMC2716485

[39] Rupaimoole R, Calin GA, Lopez-Berestein G, Sood AK. miRNA Deregulation in Cancer Cells and the Tumor Microenvironment. Cancer Discov. 2016; 6:235–46. 10.1158/2159-8290.CD-15-0893 26865249 PMC4783232

[40] Zhang H, Fan Q. MicroRNA-205 inhibits the proliferation and invasion of breast cancer by regulating AMOT expression. Oncol Rep. 2015; 34:2163–70. 10.3892/or.2015.4148 26239614

[41] Li Y, Li H, Duan Y, Cai X, You D, Zhou F, Yang C, Tuo X, Liu Z. Blockage of TGF- α Induced by Spherical Silica Nanoparticles Inhibits Epithelial-Mesenchymal Transition and Proliferation of Human Lung Epithelial Cells. Biomed Res Int. 2019; 2019:8231267. 10.1155/2019/8231267 30906781 PMC6398060

