# Supplementary material for: MiR-205 suppressed the malignant behaviors of breast cancer cells by targeting CLDN11 via modulation of the epithelial-to-mesenchymal transition
Source: Aging (Albany NY). 2021 May 8;13(9):13073–86. doi: 10.18632/aging.202988 (PMC8148491; doi:10.18632/aging.202988)
Supplement: Supplementary Figures [file aging-13-202988-s001.pdf]

SUPPLEMENTARY FIGURES

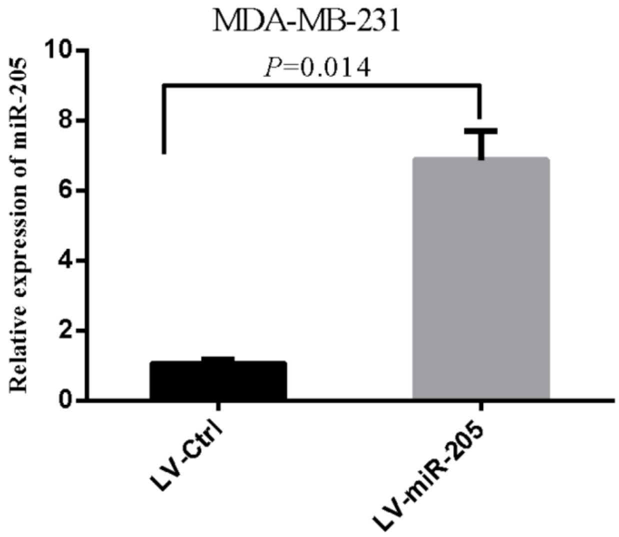

Supplementary Figure 1. miR-205 expression levels were significantly over-expressed in in breast cancer cells MDA-MB-231 infected with lentivirus-miR-205.

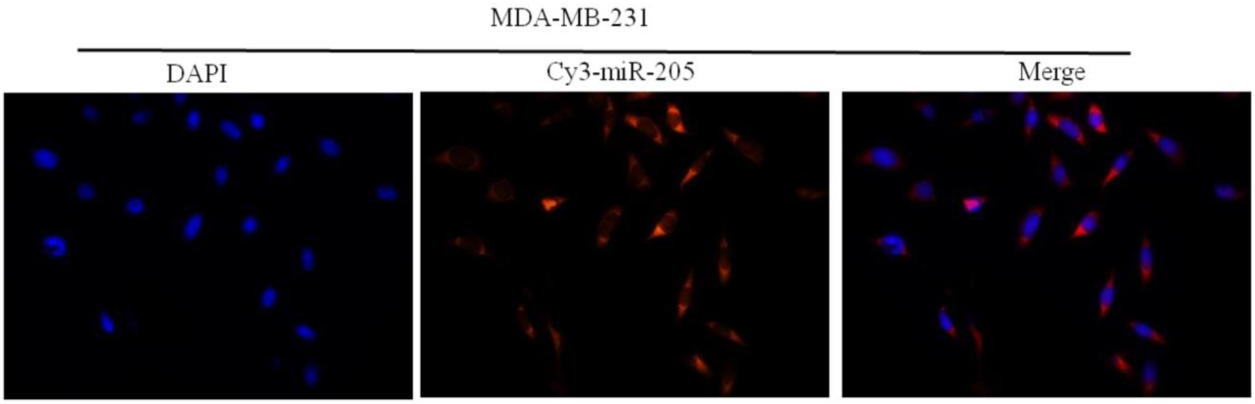

Supplementary Figure 2. FISH was used to detect miR-205 location in breast cancer cells MDA-MB-231. blue, DAPI; Red, miR-205.
